# Calycosin, a Common Dietary Isoflavonoid, Suppresses Melanogenesis through the Downregulation of PKA/CREB and p38 MAPK Signaling Pathways

**DOI:** 10.3390/ijms23031358

**Published:** 2022-01-25

**Authors:** Kun-Chang Wu, You-Cheng Hseu, Yu-Ching Shih, Govindan Sivakumar, Jyun-Ting Syu, Guan-Lin Chen, Meng-Tien Lu, Po-Chen Chu

**Affiliations:** 1 School of Pharmacy, College of Pharmacy, China Medical University, Taichung 406040, Taiwan; kunchangwu@mail.cmu.edu.tw; 2 Department of Cosmeceutics and Graduate Institute of Cosmeceutics, China Medical University, Taichung 406040, Taiwan; ychseu@mail.cmu.edu.tw (Y.-C.H.); u106044002@cmu.edu.tw (Y.-C.S.); syujyunting@gmail.com (J.-T.S.); pisceschen860320@gmail.com (G.-L.C.); tina5073300@gmail.com (M.-T.L.); 3 Drug Development Center, China Medical University, Taichung 406040, Taiwan; siva.govindan@gmail.com

**Keywords:** calycosin, melanin, tyrosinase, MITF, TRP-2, PKA/CREB, p38 MAPK

## Abstract

Calycosin, a bioactive isoflavonoid isolated from root extracts of *Astragalus membranaceus*, has been reported to inhibit melanogenesis, the mechanism of which remains undefined. In this study, we interrogated the mechanistic basis by which calycosin inhibits melanin production in two model systems, i.e., B16F10 melanoma cells and zebrafish embryos. Calycosin was effective in protecting B16F10 cells from α-melanocyte-stimulating hormone (α-MSH)-induced melanogenesis and tyrosinase activity. This anti-melanogenic effect was accompanied by decreased expression levels of microphthalmia-associated transcription factor (MITF), a key protein controlling melanin synthesis, and its target genes tyrosinase and tyrosinase-related protein-2 (TRP-2) in calycosin-treated cells. Mechanistically, we obtained the first evidence that calycosin-mediated MITF downregulation was attributable to its ability to block signaling pathways mediated by cAMP response element-binding protein (CREB) and p38 MAP kinase. The protein kinase A (PKA) inhibitor H-89 and p38 inhibitor SB203580 validated the premise that calycosin inhibits melanin synthesis and tyrosinase activity by regulating the PKA/CREB and p38 MAPK signaling pathways. Moreover, the in vivo anti-melanogenic efficacy of calycosin was manifested by its ability to suppress body pigmentation and tyrosinase activity in zebrafish embryos. Together, these data suggested the translational potential of calycosin to be developed as skin-lightening cosmeceuticals.

## 1. Introduction

The past decade has witnessed a growing interest in identifying natural anti-melanogenic or skin-lightening agents from medicinal herbs based on safety considerations [1,2]. These efforts have led to the characterization of a series of natural products, including carotenoids and polyphenols, concerning their abilities to suppress melanogenesis by blocking melanin production [2,3,4]. To date, although numerous natural compounds have been proposed to reduce pigmentation, the associated mechanism of action remains to be elucidated.

Melanin, composed of black-to-brown eumelanin and yellow-to-reddish pheomelanin, is synthesized within the melanosomes of epidermal melanocytes, whose functions are to determine skin and hair color and to defend skin against ultraviolet (UV) radiation [5]. The process of melanin synthesis consists of a series of enzymic and biochemical reactions. Tyrosinase, tyrosinase-related protein-1 (TRP-1), and tyrosinase-related protein-2 (TRP-2) are three critical enzymes involved in melanogenesis and the expression levels of these melanogenic enzymes are transcriptionally regulated mainly by the microphthalmia-associated transcription factor (MITF) in melanocytes [6,7,8]. From a translational perspective, MITF, tyrosinase, TRP-1, and TRP-2 are considered as potential targets for the development of skin-lightening cosmetics. Tyrosinase, the essential and rate-limiting enzyme in melanogenesis, catalyzes the hydroxylation of L-tyrosine to 3,4-dihydroxyphenylalanine (DOPA) and the oxidation of DOPA to DOPAquinone [9], which acts as the substrate for the synthesis of eumelanin. Similarly, TRP-1 and TRP-2 also play a crucial role in catalyzing eumelanin synthesis. In addition, DOPAquinone reacts with cysteine to form cysteinyl DOPAs, which are further oxidized and polymerized to produce yellow-to-red soluble pheomelanin [10].

Melanogenesis is a complex process, which is modulated by an array of exogenous and cellular stimuli, including UV irradiation, inflammatory cytokines, hormonal signaling, and a number of intracellular signaling pathways. For example, in response to UV exposure, keratinocytes release α-melanocyte-stimulating hormone (α-MSH) that stimulates melanin synthesis in epidermal melanocytes by activating the cyclic adenosine monophosphate (cAMP)-dependent protein kinase A (PKA)-cAMP response element-binding protein (CREB) signaling pathway [11]. Melanocortin 1 receptor (MC1R), also known as melanocyte-stimulating hormone receptor (MSHR), is a melanocytic G protein-coupled receptor that activates adenylate cyclase upon binding to α-MSH and results in increasing intracellular levels of cAMP and subsequently activating cAMP-dependent protein kinase (PKA) [12]. Activated PKA facilitates phosphorylating activation of CREB, leading to increased MITF transcriptional activity, which, in turn, stimulates the gene expressions of tyrosinase, TRP-1, and TRP-2 [13].

Along with the α-MSH-MC1R signaling pathway, increased levels of cAMP can also activate Mitogen-activated protein kinases (MAPK), including p38 MAPK, extracellular signal-regulated kinase (ERK), and c-Jun N-terminal kinase (JNK), to affect melanogenesis by regulating MITF expression [14,15]. Moreover, it has been shown that the cAMP-dependent activation of MITF and upregulation of melanogenesis can be caused by the inhibition of phosphatidylinositol 3-kinase (PI3K)/Akt signaling pathway [16,17]. Additionally, the WNT/β-catenin pathway can also contribute to MITF expression and induce melanogenesis [18].

**Figure 1:**
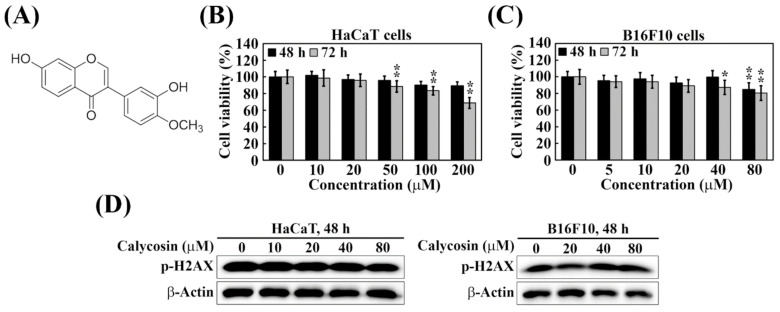
Cytotoxicity of calycosin in HaCaT and B16F10 cells. (**A**) Chemical structure of calycosin. (**B**) Human keratinocyte HaCaT cells and (**C**) mouse melanoma B16F10 cells were treated with indicated concentrations of calycosin for 48 and 72 h. Cell viability was analyzed by MTT assay. Data are represented as the means ± S.D. from three independent experiments. Significant difference versus control: * *p* < 0.05, ** *p* < 0.01. (**D**) Western blotting analysis of p-H2AX expression in HaCaT (left) and B16F10 (right) cells treated with indicated concentrations of calycosin for 48 h.

Calycosin (structure shown on Figure 1A) is a major bioactive isoflavonoid isolated from the dry root extract of a common edible traditional Chinese medicine, *Astragalus membranaceus*. It is noteworthy that calycosin exhibits multiple pharmacological activities, including anti-inflammation, anti-oxidation, and anti-tumor [19,20,21]. In addition, calycosin has also been reported to suppress melanogenesis through the inhibition of tyrosinase expression at the protein level [22], of which the underlying mechanism remains unclear. Considering the translational potential of calycosin in skin lightening, we embarked on an investigation into the mechanism by which calycosin inhibits melanin synthesis in two model systems, including B16F10 melanoma cells and zebrafish embryos. We obtained evidence that the anti-melanogenic effect of calycosin was attributable to its ability to block PKA/CREB and p38 MAPK signaling pathways.

## 2. Results

### 2.1. Calycosin Shows No Cytotoxicity and Genotoxicity in B16F10 and HaCaT Cells

We first aimed to evaluate the cytotoxic effect and determine the maximum concentration applied to the future experiments of calycosin in HaCaT keratinocytes and B16F10 melanoma cells by using an MTT assay. After treatment with calycosin for 48 h or 72 h, cell viability was still above 80% at the 100 μM and 80 μM of calycosin in HaCaT (Figure 1B) and B16F10 (Figure 1C) cells, respectively. According to the International Organization for Standardization (ISO) 10993–5:2009 (Biological Evaluation of Medical Devices), cell viability higher than 80% is considered as non-cytotoxicity. Therefore, the concentrations of calycosin at 10–80 μM, which is consistent with a previous study from Kim et al. [22], were selected for the following experiments. We further evaluated the genotoxicity of calycosin by examining its effect on the phosphorylation of histone 2AX (p-Ser139H2AX, γH2AX), the key biomarker for DNA damage [23]. As shown, exposure of HaCaT and B16F10 cells to calycosin at 10–80 μM did not give rise to appreciable changes in p-H2AX levels after 48 h (Figure 1D). Together, these data suggest a desirable safety profile of calycosin suitable for cosmetic applications.

### 2.2. Calycosin Suppresses Melanin Synthesis and Tyrosinase Activity in B16F10 Cells

**Figure 2:**
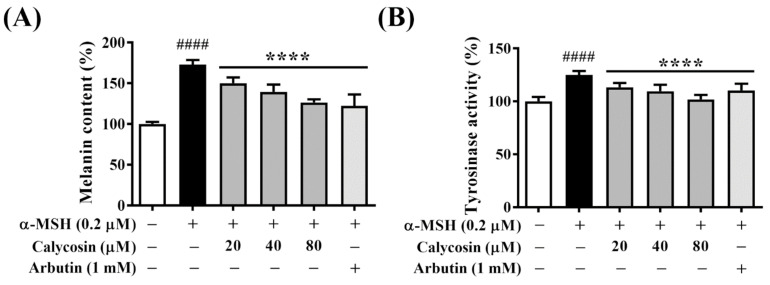
Calycosin inhibits melanin content and tyrosinase activity in B16F10 cells. Inhibitory effects of calycosin on (**A**) melanin content (%) and (**B**) tyrosinase activity (%) in B16F10 cells treated with 0.2 μM α-MSH and indicated concentrations of calycosin for 48 h. Data are represented as the means ± S.D. from three independent experiments. Significant difference versus control: #### *p* < 0.0001. Significant difference versus α-MSH-treated group: **** *p* < 0.0001. Positive control: 1 mM Arbutin.

The anti-melanogenic effect of calycosin was assessed by determining the cellular melanin content and tyrosinase activity in B16F10 cells after co-treatment with α-MSH and calycosin at 20, 40, and 80 μM versus vehicle control for 48 h. Moreover, Arbutin (1 mM), a natural product-based melanin synthesis inhibitor, was used as a positive control. As shown in Figure 2A, calycosin significantly inhibited α-MSH-induced melanin content in a dose-dependent manner. It is noteworthy that 80 μM calycosin was equipotent as 1 mM Arbutin in inhibiting melanin production (Figure 2A). In light of the critical role of tyrosinase in melanin synthesis [24], we also examined the inhibitory effect of calycosin versus Arbutin on intracellular tyrosinase activities in B16F10 cells under the aforementioned conditions. Consistent with previous evidence [22], calycosin dose-dependently suppressed α-MSH-induced tyrosinase activity (Figure 2B), paralleling the inhibitory effect on melanin production. These results clearly demonstrated the anti-melanogenic ability of calycosin, in part, through the inhibition of tyrosinase, a key melanogenic enzyme.

### 2.3. Effects of Calycosin on the Expression of Key Mediators of Melanogenesis

**Figure 3:**
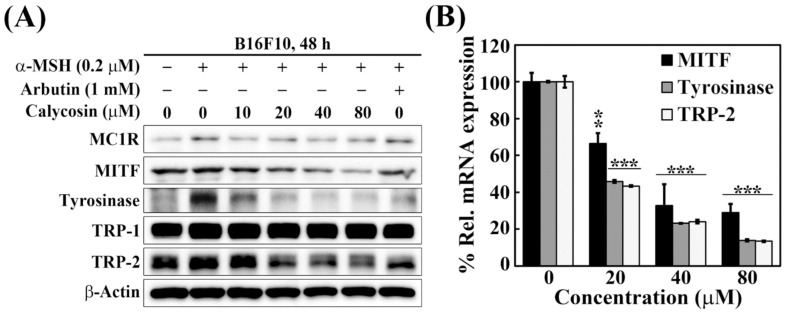
Calycosin inhibits the expressions of MITF, Tyrosinase, and TRP-2 in B16F10 cells. (**A**) Western blot analyses of the dose-dependent effect of calycosin on the expression levels of MC1R, MITF, Tyrosinase, TRP-1, and TRP-2 in B16F10 cells treated with 0.2 μM α-MSH and indicated concentrations of calycosin for 48 h. The values denote fold changes was determined by the relative intensity of protein bands of treated samples to that of the respective vehicle-treated control after normalization to the respective internal reference β-actin. (**B**) Quantitative real-time PCR analyses of the effect of calycosin on mRNA expressions of MITF, Tyrosinase, and TRP-2 in B16F10 cells treated with 0.2 μM α-MSH and indicated concentrations of calycosin for 48 h. Significant difference versus control: ** *p* < 0.01, *** *p* < 0.001.

To interrogate the role of individual melanogenic mediators by which calycosin inhibited melanin synthesis, we examined the effects of calycosin versus Arbutin on the protein expression of five key mediators, including MC1R, MITF, tyrosinase, TRP-1, and TRP-2, in α-MSH-treated B16F10 cells. While α-MSH stimulated a modest increase in the expression of these five biomarkers, calycosin could effectively downregulate the expression of MITF, tyrosinase, and TRP-2 (Figure 3A). Evidence suggested that this downregulation was mediated through transcriptional repression as real-time PCR analyses showed decreases in the mRNA expression levels of MITF, tyrosinase, and TRP-2 (Figure 3B). Together, these findings indicated that calycosin decreased the expression of MIFT at the transcriptional level, which, in turn, inhibited the gene expression of the two melanin-producing enzymes, tyrosinase and TRP-2.

### 2.4. Effect of Calycosin on the Phosphorylation of CREB, p38, JNK, ERK, and Akt

**Figure 4:**
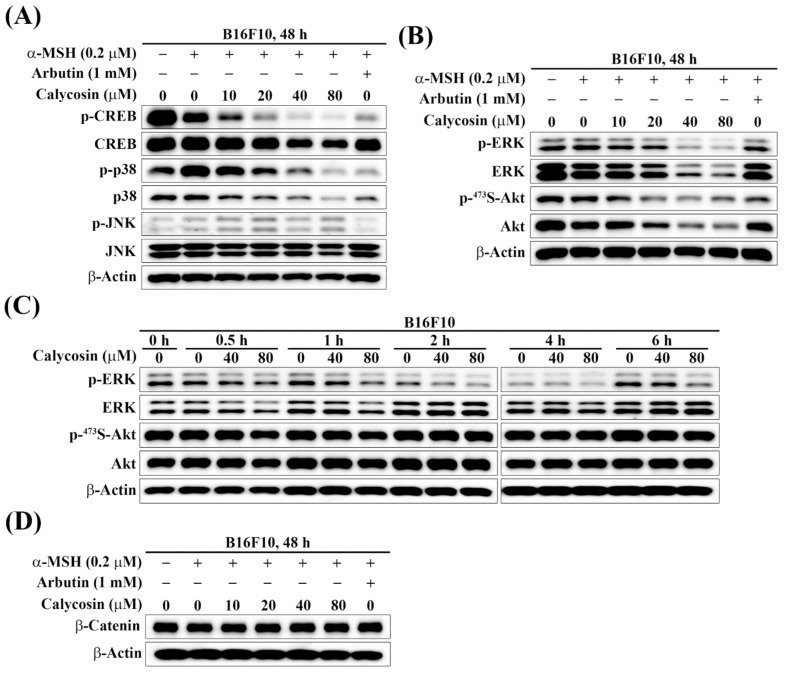
Effect of calycosin on the melanogenesis-related protein expression levels in B16F10 cells. Western blot analyses of the dose-dependent effect of calycosin on the expressions and phosphorylations of (**A**) CREB, p38, JNK, and (**B**) ERK and Akt in B16F10 cells treated with 0.2 μM α-MSH and indicated concentrations of calycosin for 48 h. (**C**) Western blot analyses of the time-dependent effect of calycosin on the expressions and phosphorylations of ERK and Akt in B16F10 cells treated with 40 μM or 80 μM calycosin for indicated time periods. (**D**) Western blot analysis of the dose-dependent effect of calycosin on the expression level of β-Catenin in B16F10 cells treated with 0.2 μM α-MSH and indicated concentrations of calycosin for 48 h.

Several signaling pathways have been implicated in α-MSH-induced melanogenesis through regulation of MITF, including those mediated by PKA/CREB, MAPK/ERK, p38 MAPK, JNK, and PI3K/Akt [11,25]. Accordingly, we systematically examined the effects of calycosin on the phosphorylation and/or expression of these potential upstream mediators to discern their involvements. Inhibition of PKA/CREB, p38 MAPK, and JNK pathways has been implicated in reducing α-MSH-induced melanogenesis through the transcriptional regulation of MITF [26,27,28]. To explore the underlying mechanisms by which calycosin inhibited MITF expression and downstream melanogenesis-related factors, we investigated whether calycosin affected PKA/CREB, p38 MAPK, and JNK signaling pathways in α-MSH-treated B16F10 cells by Western blot analysis. As shown in Figure 4A, calycosin dose-dependently inhibited the phosphorylation levels of CREB and p38; however, no appreciable changes in JNK phosphorylation levels in α-MSH-treated B16F10 cells (Figure 4A). These results indicated that calycosin suppressed expression of MITF through downregulation of PKA/CREB and p38 MAPK signaling pathways, leading to the decrease of melanogenesis.

Accumulating evidence has shown that the activation of MAPK/ERK and PI3K/Akt signaling pathways were involved in inhibiting melanogenesis [29,30,31,32]. It was reported that the activation of ERK resulted in the phosphorylation of MITF at Ser73, which induced the ubiquitin-dependent proteasomal degradation of MITF, and inhibition of melanogenesis [30]. Moreover, activation of the PI3K/Akt pathway led to the inhibition of the MITF transcriptional activity for melanogenic proteins, resulting in the suppression of melanin synthesis [32]. Therefore, we next investigated whether calycosin inhibited melanogenesis through activation of the MAPK/ERK and PI3K/Akt pathways in α-MSH-treated B16F10 cells. Western blot analyses indicated that the phosphorylation levels of ERK and Akt were decreased in calycosin-treated α-MSH-stimulated B16F10 cells for 48 h (Figure 4B), suggesting that the inhibitory effect of calycosin on melanogenesis is not associated with the MAPK/ERK and PI3K/Akt pathways. To further substantiate the effect of calycosin on the MAPK/ERK and PI3K/Akt pathways, the time courses of ERK and Akt phosphorylations were determined by Western blotting analysis in calycosin-treated B16F10 cells. As shown, no significant increase of ERK and Akt phosphorylations were detected even in short-term time course treatments of calycosin (Figure 4C), which refuted the involvement of MAPK/ERK and PI3K/Akt pathways in calycosin-mediated antimelanogenesis in B16F10 cells.

### 2.5. Effect of Calycosin on the Expression of β-Catenin

Several studies have shown that the upregulation of β-catenin in the Wnt/β-catenin signaling pathway induced the expression and transcriptional activity of MITF in melanogenesis [33,34,35]. Wnt/β-catenin signaling has recently emerged as a critical regulator participating in melanin synthesis. To evaluate whether Wnt/β-catenin signaling is involved in calycosin-mediated antimelanogenesis, β-catenin expression was examined in α-MSH-treated B16F10 cells. Western blotting analysis revealed that no significant changes of β-catenin expression were observed in calycosin-treated α-MSH-stimulated B16F10 cells (Figure 4D), indicating that Wnt/β-catenin signaling is not involved in calycosin-mediated antimelanogenesis.

### 2.6. Effect of Calycosin on Melanogenesis-Related Signaling Pathways

To further explore the molecular mechanisms underlying the inhibitory effect of calycosin on melanogenesis, the PKA inhibitor H-89, MAPK/ERK inhibitor PD98059, PI3K/Akt inhibitor LY294002, and p38 MAPK inhibitor SB203580 were used to examine the involvement of these melanogenesis-related signaling pathways in calycosin-mediated antimelanogenesis.

**Figure 5:**
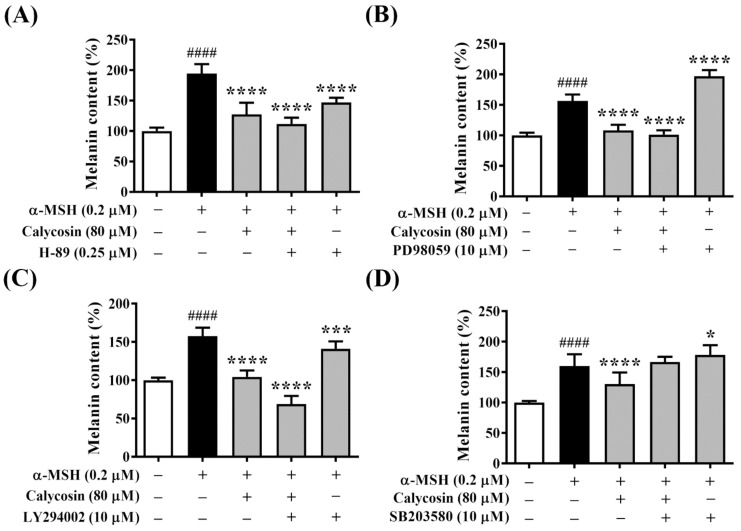
Effects of Calycosin on the melanogenesis signaling pathway. (**A**) Effect of calycosin and H-89 (PKA inhibitor) on melanin content (%) in α-MSH-treated B16F10 cells for 48 h. (**B**) Effect of calycosin and PD98059 (MAPK/ERK inhibitor) on melanin content (%) in α-MSH-treated B16F10 cells for 48 h. (**C**) Effect of calycosin and LY294002 (PI3K/Akt inhibitor) on melanin content (%) in α-MSH-treated B16F10 cells for 48 h. (**D**) Effect of calycosin and SB203580 (p38 inhibitor) on melanin content (%) in α-MSH-treated B16F10 cells for 48 h. Data are represented as the means ± S.D. from three independent experiments. Significant difference versus control: #### *p* < 0.0001. Significant difference versus α-MSH-treated group: * *p* < 0.05, *** *p* < 0.001, **** *p* < 0.0001.

PKA-mediated phosphorylation of CREB is one of the major stimulatory signals to activate MITF transcriptional activity. To determine whether the PKA/CREB pathway is involved in the inhibition of melanogenesis by calycosin, melanin content was analyzed in B16F10 cells treated with calycosin and H-89 for 48 h. As shown in Figure 5A, treatments with calycosin and H-89 reduced α-MSH-induced melanin content to 127.4% and 146.9% compared to a vehicle control treatment in B16F10 cells, respectively. Moreover, cotreatment with calycosin and H-89 significantly reduced melanin content to 111.7% compared to a vehicle control treatment (Figure 5A), suggesting that the PKA/CREB pathway was involved in the antimelanogenic effect of calycosin.

Next, to determine whether the MAPK/ERK pathway is involved in the inhibition of melanogenesis by calycosin, melanin content was analyzed in B16F10 cells treated with calycosin and PD98059 for 48 h. The melanin content was induced to 156.7% after α-MSH treatment and increased to 197.2% after cotreatment with PD98059 (Figure 5B), which supports the evidence that inhibition of the MAPK/ERK pathway increased melanogenesis [36]. However, the melanin content was reduced to 101.3% after the cotreatment with calycosin and PD98059 in α-MSH-treated B16F10 cells, which showed no significant difference from the treatment of calycosin alone (Figure 5B), suggesting that the MAPK/ERK pathway was not involved in the antimelanogenic effect of calycosin.

To determine whether inhibition of melanogenesis by calycosin was regulated by the PI3K/Akt pathway, melanin content was analyzed in B16F10 cells treated with calycosin and LY294002 for 48 h. The melanin content was induced to 157.7% and 141.2% after α-MSH treatment and cotreatment with LY294002 compared to a vehicle control treatment in B16F10 cells, respectively (Figure 5C). However, the melanin content was reduced to 68.9% after cotreatment with calycosin and LY294002 in α-MSH-treated B16F10 cells, suggesting that the PI3K/Akt pathway was not involved in the antimelanogenic effect of calycosin.

To determine whether the p38 MAPK pathway is related to the inhibitory effect of calycosin on melanogenesis in α-MSH-treated B16F10 cells, melanin content was analyzed in B16F10 cells treated with calycosin and SB203580 for 48 h. The melanin content was induced to 160.0% and 178.5% after α-MSH treatment and cotreatment with SB203580 compared to a vehicle control treatment in B16F10 cells, respectively (Figure 5D). Additionally, cotreatment with calycosin and SB203580 significantly increased melanin content to 166.7% compared to a vehicle control treatment (Figure 5D), suggesting that the p38 MAPK pathway was involved in the antimelanogenic effect of calycosin.

### 2.7. In Vivo Antimelanogenesis Effect of Calycosin

**Figure 6:**
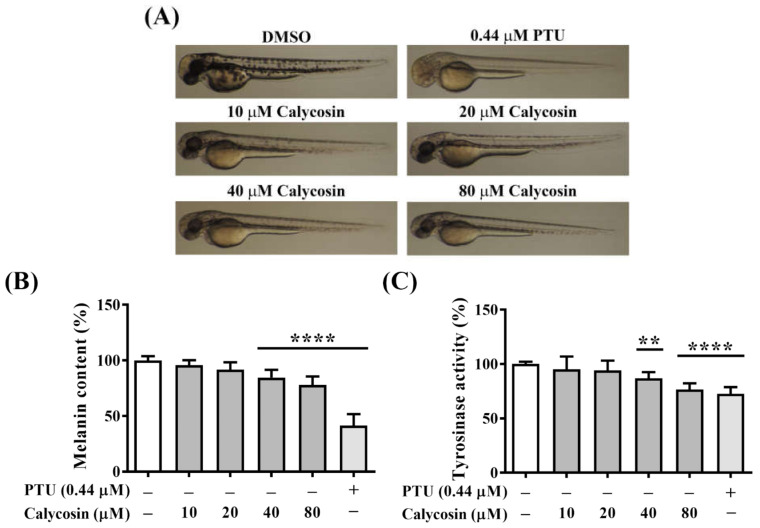
The inhibitory effect of calycosin on zebrafish pigmentation. (**A**) Representative images of melanin distribution in zebrafishes treated with indicated concentrations of calycosin. Positive control: 0.44 μM phenylthiourea (PTU). (**B**) Effects of calycosin on (**B**) melanin content (%) and (**C**) tyrosinase activity (%) in zebrafishes treated with indicated concentrations of calycosin. Data are represented as the means ± S.D. from three independent experiments. Significant difference versus DMSO control: ** *p* < 0.01, **** *p* < 0.0001.

To investigate whether calycosin has a relevant in vivo inhibitory effect on melanin synthesis, zebrafish embryos were treated with indicated concentrations of calycosin for 72 h, and the melanin content and tyrosinase activity were evaluated in developed larvae (Figure 6). Calycosin-treated embryos showed the reduced body pigment of zebrafish larvae in a dose-dependent manner compared with the vehicle-treated group (Figure 6A). Furthermore, we examined total melanin content and tyrosinase activity using whole extracts of zebrafish. There were significant decreases in total melanin content and tyrosinase activity after treatment with 40 μM and 80 μM calycosin (Figure 6B,C), indicating the potent in vivo antimelanogenic activity of calycosin in zebrafish.

## 3. Discussion

Besides providing the pigments for skin, hair, and eye color, melanin also plays the key role in protecting skin cells against ultraviolet (UV) radiation and oxidative stress [37]. Aberrant regulation of melanogenesis, either increased or decreased production of melanin, is associated with several skin disorders, such as melasma, freckles, and vitiligo [11]. Therefore, the melanogenesis in melanocyte is a tightly regulated process and can be modulated by a variety of intrinsic and extrinsic factors, leading to hyperpigmentation or hypopigmentation [38].

In the process of melanin synthesis, MITF, a basic helix-loop-helix leucine zipper (bHLH-ZIP) transcription factor, plays a critical role in regulating the expressions of several melanogenesis-related enzymes, such as tyrosinase, TRP-1, and TRP-2. In addition to pigmentation enzymes, MITF is also the main transcriptional regulator of many genes for melanocyte development, differentiation, and melanosome trafficking [7]. The expression of MITF is regulated by several signaling pathways in α-MSH-induced melanogenesis. The binding of α-MSH to the MC1R receptor activates adenylyl cyclase activity, which triggers intracellular cAMP production, followed by the activation of PKA/CREB and three major MAPK pathways, including JNK, MAPK/ERK, and p38 MAPK [25]. Activation of the PKA/CREB, JNK, and p38 MAPK pathways has been reported to induce the expression of MITF, tyrosinase, and TRPs, leading to melanogenesis [39].

*Astragalus membranaceus* has a long history of medical use in Chinese herbal medicine [40], and is widely used in the treatment of inflammatory diseases, tumors, cardiovascular diseases, and neuroprotective diseases [22]. Triterpene saponins, flavonoids, and polysaccharides are major active constituents in *Astragalus membranaceus* [41]. Many flavonoid compounds, such as EGCG, hesperidin, baicalein, and quercetin, derived from natural products possess potent antioxidant and antimelanogenic activities [42,43,44,45]. For instance, hesperidin and baicalein, two well-known antioxidants, inhibited melanogenesis by activating ERK phosphorylation which subsequently degraded MITF resulting in the suppression of melanogenic enzymes and melanin synthesis. In addition to flavonoids, a previous study has demonstrated that the polysaccharide isolated from *Astragalus membranaceus* also exhibited an inhibitory effect on melanogenesis by activating ERK phosphorylation in α-MSH-treated B16F10 cells [46].

**Figure 7:**
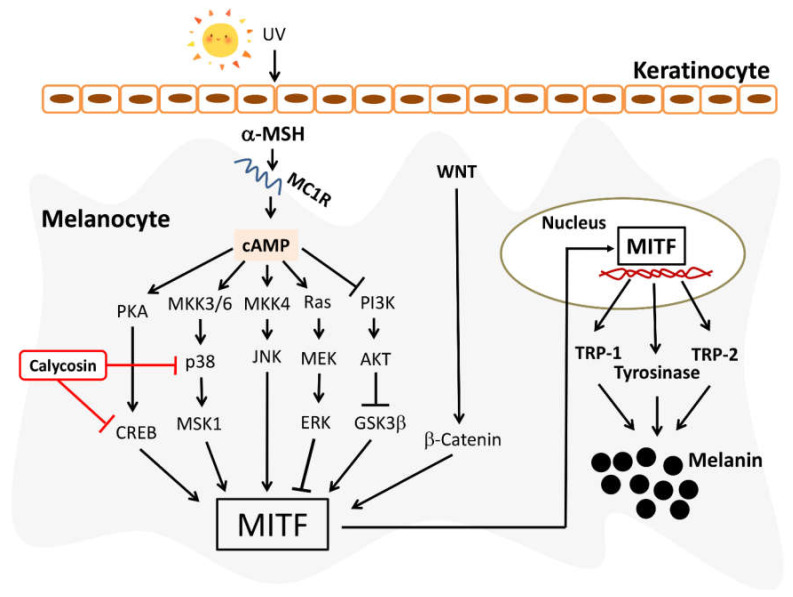
Proposed regulatory mechanisms of calycosin on suppression of melanin synthesis in melanocyte. The black arrows indicate stimulating signals and the red T bars indicate inhibitory effects.

Calycosin, one of the major bioactive isoflavonoids of *Astragalus membranaceus*, exhibits promising effects for the treatment of several diseases and has been used as the representative bioactive chemical of *Astragalus membranaceus* [19]. A previous study has reported that calycosin suppressed melanin production by inhibiting tyrosinase expression in Melan-a murine melanocytes [22]. However, the underlying molecular mechanisms for the antimelanogenic effect of calycosin remains undefined. In this study, we showed that calycosin significantly inhibited α-MSH-induced melanogenesis and melanin-related gene expression, including MITF, tyrosinase, and TRP-2, in B16F10 cells. However, no significant change of TRP-1, one of the target genes of MITF, was found after calycosin treatment, which warrants further investigation. It is noteworthy that the inhibitory potency of calycosin on melanin and tyrosinase were higher than the well-known skin-whitening agent Arbutin (Figure 2). Furthermore, we also unveiled the possible molecular mechanisms by which calycosin inhibited melanogenesis with the inhibitors of the PKA/CREB, MAPK/ERK, PI3K/Akt, and p38 MAPK signaling pathways. Mechanistically, our results indicated that calycosin inhibited melanin synthesis by regulating PKA/CREB and p38 signaling pathways to suppress MITF expression, as well as MITF downstream enzymes, tyrosinase, and TRP-2 in B16F10 cells (Figure 7). In addition, calycosin notably reduced the in vivo body pigment of zebrafish larvae with no adverse side effects. In conclusion, this study demonstrated the antimelanogenic activity and underlying mechanism of calycosin and suggested that calycosin could be used as a skin-lightening agent for the cosmetic products.

## 4. Material and Methods

### 4.1. Cell Culture, Chemicals, and Antibodies

HaCaT human keratinocytes were kindly provided by Dr. Louis-kuoping Chao. B16F10 mouse melanoma cells were purchased from the Bioresource Collection and Research Center (BCRC, Hsinchu, Taiwan). Both cell lines were maintained in Dulbecco’s Modified Eagle’s Medium (DMEM) (GIBCO, Grand Island, NY, USA) with 10% fetal bovine serum (Thermo Fisher Scientific, Carlsbad, CA, USA) at 37°C in a humidified incubator containing 5% CO_2_. Calycosin was synthesized by Small Molecule Medicinal Chemistry Laboratory in China Medical University. L-DOPA, Arbutin, and α-MSH were obtained from Sigma-Aldrich (St. Louis, MO, USA); H-89, PD98059, LY294002, SB203580, SB216763, and Propylthiouracil (PTU) were obtained from Cayman Chemical (Ann Arbor, MI, USA). Antibodies used in this study and their sources were as follows: β-actin, MC1R, MITF, Tyrosinase, TRP-1, TRP-2, p38, p-p38, β-Catenin (Santa Cruz Biotechnology, Santa Cruz, CA, USA); p-H2AX Ser139 (Millipore, Billerica, MA, USA); JNK, p-JNK, CREB, p-CREB, ERK, p-ERK, Akt, p-Akt Ser473, GSK3β, p-GSK3β Ser9 (Cell Signaling, Beverly, MA, USA).

### 4.2. Cell Viability Assay

The effect of calycosin on cell viability were determined by 3-(4,5-dimethylthiazol-2-yl)-2,5-diphenyltetrazolium bromide (MTT) assays. Cells were seeded in 96-well plates at a density of 5000 cells per well in the presence of 10% FBS. After overnight incubation, cells were exposed to calycosin, which is freshly prepared in DMSO and diluted with culture medium, at the indicated concentrations with the final DMSO concentration at 0.1% vis-à-vis vehicle (0.1% DMSO-containing medium) for 48 or 72 h. After treatment, cells were incubated with MTT (Biomatik, Wilmington, DE, USA) for an additional 1 h. The medium was then removed from each well and replaced with DMSO to dissolve the MTT dye for subsequent colorimetric measurement of absorbance at 570 nm. Cell viabilities were expressed as percentages of viable cells relative to the corresponding vehicle-treated control group.

### 4.3. Melanin Content Assay

Melanin content was measured according to a previously described method with slight modification [47]. Briefly, B16F10 cells (2.5 × 10^4^ cells/well) were seeded into 24-well plates in the culture medium for 24 h. After 24 h incubation, cells were treated with various concentrations of calycosin for 48 h and α-MSH (200 nM) was used as the positive control treatment. After treatment, cells were lysed by adding 2 N NaOH to each well and the supernatants were collected by centrifugation. The amounts of melanin in the supernatant were spectrophotometrically measured at 405 nm. The melanin content in the treated cells were expressed as percentages relative to the corresponding vehicle-treated control cells.

### 4.4. Cellular Tyrosinase Activity Assay

Cellular tyrosinase activity was determined using a previously described method with modification [48]. The B16F10 cells were plated in 24-well plates and treated with a medium containing α-MSH (200 nM) and various concentrations of calycosin. After 48 h, the medium was removed, and phosphate-buffered saline with 1% Triton X-100 was added to each well. The mixture was frozen at −80°C and thawed at room temperature, and then centrifuged to obtain the supernatant from the wells. The freshly prepared substrate (15 mM L-DOPA) was added to the supernatant and the absorbance was subsequently read at 475 nm.

### 4.5. Immunoblotting

Cell pellets were lysed in SDS lysis buffer and sonicated, and the protein concentration of each sample was determined by using the BCA Protein Assay kit (Thermo Fisher Scientific). An equal amount of protein from each sample was loaded per lane, separated by SDS-PAGE, and then transferred onto nitrocellulose membranes. Transferred membranes were blocked with 5% non-fat milk for 1 h and then incubated with primary antibodies overnight at 4°C. On the next day, membranes were washed with TBST and incubated with the corresponding secondary antibodies for 1h at room temperature. Chemiluminescence Reagent Plus (Perkin-Elmer; Waltham, MA, USA) was used to detect signals.

### 4.6. Quantitative Real-Time PCR (qRT-PCR)

Total RNA was isolated and reverse transcribed to cDNA using a RNeasy mini kit (Qiagen, Hilden, Germany) and TOOLS Easy Fast RT kit (Biotools, Taipei, Taiwan), respectively, according to the manufacturer’s instructions. qRT-PCR was carried out using the StepOne system with Fast SYBR Green Master Mix (Thermo Fisher Scientific) and the following primers: MITF: 5′-ACTTTCCCTTATCCCATCCACC-3′ and 5′-TGAGATCCAGAGTTGTCGTACA-3′; Tyrosinase: 5′-CTCTGGGCTTAGCAGTAGGC-3′ and 5′-GCAAGCTGTGGTAGTCGTCT-3′; TRP-2: 5′-GTCCTCCACTCTTTTACAGACG-3′ and 5′-ATTCGGTTGTGACCAATGGGT-3′; 18s ribosome RNA: 5′-ACCCGTTGAACCCCATTCGTGA-3′ and 5′-GCCTCACTAAACCATCCAATCGG-3. Relative gene expression was normalized to 18s rRNA and calculated by using the 2^−^^ΔΔCt^ method. Student’s *t*-test was performed to compare treated and control groups with significance reported for *p*  <  0.05.

### 4.7. Melanin Contents of Zebrafish

The zebrafish experiments were performed in accordance with the relevant guidelines and regulations and approved by the Institutional Animal Care and Use Committee (IACUC) of China Medical University. The zebrafish were maintained in the 5 L glass tanks at 28°C with circulating systems that continuously filter and aerate the water to maintain the water quality and with a consistent 14:10 h light–dark cycle. Zebrafish were fed three times a day with live brine shrimps. Before the day to collect embryos, we transferred the adult zebrafish (at least 8 months of age) with the ratio of 3 males to 6 females to opposite sides of the breeding tank. We removed the divider the next morning shortly after the onset of light and allowed mating to occur undisturbed until enough numbers of embryos were laid at the bottom of the tank. The embryos were collected and arrayed by 3 mL droppers.

The melanin contents of zebrafish were determined based on the previously reported procedure with minor modifications [49]. Briefly, about 50 zebrafish embryos were treated with indicated compounds for 72 h and then sonicated in RIPA buffer. After centrifugation, the pellet was dissolved in 0.7 mL of 1 N NaOH at 60°C for 1 h. The absorbance of the supernatant was measured at 405 nm, and the result was compared with the DMSO control which was considered to represent 100%. The melanin content was calibrated by protein amounts, and the results were obtained from three independent experiments.

### 4.8. Statistical Analysis

Most of the results in this study are presented as means ± standard deviations (S.D.). Most of the statistical analyses were performed using GraphPad Prism version 8 (Graphpad, San Diego, CA, USA). A comparison between different groups was carried out using one-way analysis of variance (ANOVA), followed by Tukey’s multiple comparison tests. The data of cell viability and real-time PCR analysis were statistically compared using Student’s *t*-test. The difference is considered significant when *p* < 0.05.

## 5. Conclusions

This study demonstrated the anti-melanogenic activity of calycosin, a common dietary isoflavonoid, by decreasing expressions of MITF and its target genes tyrosinase and TRP-2 in B16F10 melanoma cells. Calycosin exhibited anti-melanogenic activity through the regulation of PKA/CREB and p38 MAPK-mediated MITF downregulation, resulting in the suppression of melanin synthesis. Although further clinical studies are required to prove the effectiveness of calycosin, our study has provided valuable information to aid the development of calycosin as potential skin-lightening cosmeceuticals.

## Data Availability

Not applicable.

## Abbreviations

α-MSH	α-melanocyte-stimulating hormone
MC1R	melanocortin 1 receptor
MITF	microphthalmia-associated transcription factor
TRP-1	tyrosinase-related protein-1
TRP-2	tyrosinase-related protein-2
cAMP	cyclic adenosine monophosphate
PKA	protein kinase A
MAPK	mitogen-activated protein kinase
JNK	Jun N-terminal kinas
CREB	cAMP response element-binding protein
GSK3β	glycogen synthase kinase 3 beta
